# Investigating the Effect of Personality, Internet Literacy, and Use Expectancies in Internet-Use Disorder: A Comparative Study between China and Germany

**DOI:** 10.3390/ijerph15040579

**Published:** 2018-03-23

**Authors:** Benjamin Stodt, Matthias Brand, Cornelia Sindermann, Elisa Wegmann, Mei Li, Min Zhou, Peng Sha, Christian Montag

**Affiliations:** 1 General Psychology: Cognition and Center for Behavioral Addiction Research (CeBAR), University of Duisburg-Essen, 47057 Duisburg, Germany; benjamin.stodt@uni-due.de (B.S.); matthias.brand@uni-due.de (M.B.); elisa.wegmann@uni-due.de (E.W.); 2 Erwin L. Hahn Institute for Magnetic Resonance Imaging, 45141 Essen, Germany; 3 Department of Molecular Psychology, Institute of Psychology and Education, Ulm University, 89081 Ulm, Germany; cornelia.sindermann@uni-ulm.de; 4 Student Counselling Centre, Beijing University of Civil Engineering and Architecture, Beijing 100037, China; amorelm415@gmail.com; 5 Institute of Medical Statistics, Informatics and Epidemiology, University of Cologne, 50923 Cologne, Germany; min.zhou@hotmail.de; 6 School of Journalism and Communication, Southwest University, Chongqing 400716, China; a2352893@gmail.com; 7 Key Laboratory for NeuroInformation/Center for Information in Medicine, School of Life Science and Technology, University of Electronic Science and Technology of China, Chengdu 611731, China

**Keywords:** Internet addiction, Internet-use disorder, Internet literacy, expectancies, personality, cultural differences

## Abstract

Research on Internet-use Disorder (IUD) has increased rapidly, indicating its clinical and global importance. Past studies suggested cultural diversity regarding the prevalence of an IUD, e.g., between Asian and European countries. Additionally, it was found that personality factors, Internet-related cognitions and specific competences seem to influence IUD tendencies, but research lacks in cultural comparative studies regarding these mechanisms. This study focuses on differences between Germany and China regarding the above-mentioned characteristics. German (*n* = 411; *M* = 20.70 years, *SD* = 3.34 years) and Chinese participants (*n* = 410; *M* = 20.72 years, *SD* = 2.65 years) answered the short Internet Addiction Test, Big Five Inventories, the Internet-use Expectancies Scale, as well as the Internet Literacy Questionnaire. The results revealed higher occurrence of IUD symptoms in China. Furthermore, Chinese participants scored significantly higher on neuroticism and agreeableness, whereas German participants scored higher on extraversion and openness. Compared to German participants, Chinese showed higher expectancies to avoid negative feelings online and to be positively reinforced. Regarding Internet literacy, German participants indicated higher skills concerning the reflection and critical analysis of online content, whereas Chinese showed higher expertise in producing and interacting online. Further, simple slope analyses indicated that certain Internet literacy domains were related differentially to IUD symptoms in Germany and China. While Chinese participants with higher reflective skills indicated highest IUD symptoms, reflective skills revealed no effect in Germany. Additionally, higher self-regulative skills correlated with lower IUD symptoms in the German, but not in the Chinese sample. The results give a hint to potential cultural differences regarding IUD, especially on the predictive and protective role of Internet literacy domains.

## 1. Introduction

Since the late 90s, the phenomenon of Internet addiction or Internet-use disorder (IUD) is focused more and more in the scientific literature (based on the term Internet-gaming disorder, the term Internet-use disorder is used in the following as a synonym for Internet addiction or pathological Internet use). Further, the number of theoretical assumptions about its classification as well as empirical studies, which give insights into the underlying mechanisms of its development, increase constantly [1,2,3]. Past research differentiates between unspecific and specific IUDs [4]. While an unspecific IUD goes along with an excessive and uncontrolled use of different Internet applications and websites without a clear preference [1], a specific IUD describes the addictive use of one specific application or certain type of website, such as games, shopping sites, or Internet communication applications (for an overview, see [2]). Not just since Internet-gaming disorder has been added to section III of the DSM-5 [5], there is an undisputable clinical relevance of IUDs. Nevertheless, further research is needed to conduct and clarify potential diagnostic criteria for an unspecified IUD or further specific forms, such as Internet-pornography-use disorder or Internet-communication disorder (ICD).

### 1.1. Prevalence of an IUD

According to a meta-analysis by Cheng and Li [6], who reviewed studies from 31 nations across seven world regions, the global occurrence of an IUD is estimated at 6%, illustrating it as a global phenomenon and universal issue. Past research on the epidemiology and cultural diversity of an IUD showed that prevalence rates strongly differ between different countries and cultures [7]. Cheng and Li [6] reported an occurrence of IUD symptoms in 2.6% of European individuals, which confirms the results by Spada [8], who identified 1 to 9% of the European population showing symptoms of a pathological Internet use. In a German representative population sample, a prevalence rate of 1% was estimated [9]. While prevalence rates in the Middle East range between 1% and 12%, highest prevalence rates up to 38% are reported for Asian countries [7,8,10]. Confirming the quality of real life hypothesis, Cheng and Li [6] identified several environmental characteristics associated with a higher occurrence of an IUD, like perception of less life satisfaction, greater traffic commute time consumption, as well as lower national income. Other reasons for cultural differences, such as Hofstede’s [11] assumptions on power-distance, collectivism, and individualism, have also been discussed [12]. Besides, the broad range of reported prevalence rates can also be due to the absence of standardized diagnostic tools. Former studies used various questionnaires to measure symptoms of an IUD, which are grounded on different diagnostic criteria and cut-off scores [7]. Consequently, cross-cultural differences in the prevalence of an IUD found in previous studies still require a cautious interpretation. 

### 1.2. Cultural Issues of an IUD: Results from Germany and China

Along with research on the occurrence of an IUD, former studies already focused on specific psychological aspects which are associated with an excessive and addictive use of the Internet in different nations and cultures [7,13,14]. However, there are just a few studies concentrating on cultural issues regarding the occurrence of an IUD as well as the effect of personality traits and Internet-related state variables on its development. In order to better understand the development and maintenance of an IUD within different national backgrounds, cross-cultural studies are important [13]. Besides, Ko and Yao [15] emphasized that cultures psychologically differ in a variety of individual characteristics, such as personality, emotions, and cognitions. Therefore, nationally differing effects of personal characteristics on the development of an IUD can be expected.

Within the scope of cultural-comparative studies, Asian and Western samples have been compared most frequently regarding specific individual characteristics as well as economic and infrastructural background [15]. Regarding digitization and Internet penetration rates, Germany and China display two key nations of their continents [16,17]. In Germany, 89.6% of the population is online (which are 72.3 million people) [16]. This corresponds to 11.1% of all European Internet users and is the second highest rate after Russia (16.6%). Nevertheless, only 76.4% of the Russian population is online [16]. In China, the Internet penetration rate is at 54.6% (772 million users), which displays the highest amount of Internet users in Asia with 38.1% in terms of total user numbers [16]. The highest Internet penetration rate for Asian nations is found in Japan (93.3%) [16]. However, this rate corresponds to just 5.9% of the Asian population. Due to these facts, we chose Germany and China as two representatives for well-digitized European and Asian nations.

Within Germany, IUD displays a serious health problem. In a representative study, 1% of the German population has been identified as pathological Internet users [9]. Higher prevalence rates were observed within the age of 14 to 24 (2.4%) and 14 to 16 years (4%) [9]. Another German study reported a prevalence rate of 2.1%, where the group of people suffering from IUD reported significant psychosocial and health consequences due to their excessive Internet use [18]. Further, male gender and social factors were significantly associated with IUD symptoms [18]. Further studies from Germany highlighted significant correlations of tendencies towards an unspecific and specific IUD with psychopathological symptoms (e.g., depression and interpersonal sensitivity [19,20,21]), higher impulsivity [19], younger age [22] and male gender [18,19].

Even if IUD is a growing topic of research in Germany, IUD has been studied more frequently in China. There, IUD has been recognized as a nationwide health problem with prevalence rates ranging from 2.4 to 6.4% [23,24]. Within China, Internet addicted individuals have been characterized with higher impulsivity [25], lower prosocial behaviors [23], higher depressive symptoms [24,26], shyness [27], lower self-esteem [26], younger age [24,28], and male gender [28]. 

In the following, results on the effect of specific personality traits and Internet-related state variables on IUD symptoms are summarized. Here, we focus on investigations carried out in Germany and China, supplemented by results from Western and Asian countries.

### 1.3. Predictors of an IUD: Trait Variables

Beyond epidemiological studies, many studies focus on individual factors influencing the development and maintenance of IUDs. Here it has to be distinguished between personal predispositions [29,30], which persist over a long period of time (traits), and factors which are situation-dependent, such as expectancies or actual mood (states) [31,32]. Regarding trait variables, depression, attention-deficit/hyperactivity disorder (ADHD), as well as social anxiety disorders have been verified as three main comorbid personal predispositions of IUDs [32,33,34]. Next to these predispositions, further person’s core characteristics could play an important role in the addiction process. One popular concept of one person’s core personality is the Big Five personality model, including the personality traits of neuroticism, extraversion, openness to experience, conscientiousness, and agreeableness [35,36]. In their theoretical model, called I-PACE, Brand et al. [2] describe different stages of the addiction process of specific Internet-use disorders, including the interaction of state and trait variables as well as different mediation effects. In this model and their previous literature review, the authors highlighted the relevance of the Big Five personality traits, especially low conscientiousness, low extraversion, and high neuroticism, as predispositions in persons who are vulnerable to use specific Internet applications excessively and uncontrolled [2].

According to cross-cultural studies, differences regarding the Big Five personality traits between East Asian and Western cultures exist. A meta-analysis revealed that especially East Asian countries score significant lower on the traits of extraversion, agreeableness, conscientiousness, and openness, but higher on neuroticism than other countries and cultures [37]. Moreover, mainly higher neuroticism and lower extraversion were found as main correlates of an IUD across different countries [38,39,40]. However, openness, conscientiousness and agreeableness also indicated significant relations to IUD symptoms within a sample of Chinese adolescents [28]. Despite these general cultural differences concerning Big Five personality traits and the correlations with IUD symptoms found within country-specific samples, it remains under-uninvestigated whether the strength of impact regarding these traits on the development and maintenance of an IUD significantly differs between different countries and cultures. 

### 1.4. Predictors of an IUD: State Variables

Next to personal predispositions and traits, researchers focused on state variables, which are considered as situation-dependent factors, influencing and triggering human behavior [31]. In the context of an IUD, such factors are especially motivational factors, coping strategies, and cognitive biases [2,3]. It is assumed that these factors interact with trait variables and core characteristics in the developmental process of an IUD and in turn reinforce and stabilize cognitive and affective responses [2]. In the following, Internet-use expectancies and specific Internet literacy domains are discussed as two exemplary predictors of an IUD.

#### 1.4.1. Internet-Use Expectancies

According to Lee et al. [41], person’s expectancies towards the use of the Internet affect his or her attitudes regarding specific online applications. Furthermore, Internet users’ expectancies (e.g., to gratify a certain need) lead to the decision to use the Internet or a specific application to experience gratification, as shown in the I-PACE model by Brand et al. [2] (also see their article for a more comprehensive overview of this decision-making process). One step further, symptoms of an IUD increase, when positive expectancies and metacognitions towards the Internet exist [42,43]. Brand et al. [44] already showed that Internet-use expectancies for positive reinforcement as well as avoidance expectancies are correlated with psychopathological symptoms and further personality aspects within a German sample. In turn, certain use expectancies and dysfunctional coping mechanisms are correlated with symptoms of an unspecified IUD. Also within a German sample, Wegmann et al. [21] could replicate these findings, as they found associations between psychopathological symptoms, Internet-use expectancies, self-regulation abilities, and symptoms of an excessive use of Internet-communication applications. According to a Taiwanese study by Lee et al. [41], Internet-use expectancies positively predicted students’ attitudes towards and the use of specific Internet applications. In another Taiwanese study, Wu et al. [45] found that having expectancies that the use of the Internet helps to relieve negative emotions increases the probability of IUD symptoms in persons with borderline personality disorder. The authors stated that it is important to teach individuals to develop alternative coping strategies and not to use the Internet for escaping from negative feelings.

#### 1.4.2. Internet Literacy

As an additional state variable, specific skills and competences in using the Internet and its applications could also play a key role in the development and maintenance of IUDs [22]. Today, there is still no common term summarizing such skills, whereas researchers stated that more empirical research in this area is required [46]. Some of these concepts are termed e.g., digital literacy [47], new media literacy [48,49], information literacy [50], or Internet literacy [22,51]. Fraillon et al. [52] defined digital literacy as “an individual’s ability to use computers to investigate, create, and communicate in order to participate effectively at home, at school, in the workplace, and in society” (p. 18). Stodt, Wegmann, and Brand [22] recently published a four-dimensional concept of Internet literacy which they had also operationalized with a self-assessment questionnaire. Beyond the pure technical expertise in handling Internet applications and relevant software, the concept of Internet literacy by Stodt et al. [22] specifically focuses on a rather deliberate, conscious and reflective use of the Internet and certain applications (see also [51]). Besides that, the increasing possibilities through interactive participation brought by social media cause that Internet users need to critically evaluate online content and information. This is also crucial to become an inherent part of online social networks as an active producer of content [53,54]. As a last skill, it is necessary for individuals to control and self-regulate their own online behavior [55].

Some empirical studies found that specific domains of an Internet literacy could be both positively and negatively related to symptoms of an IUD or ICD in Asian as well as European samples, e.g., [22,51]. More detailed, the high impact of self-regulative skills in IUD and ICD was already highlighted in Western cultures, e.g., [21,56]. Here, the authors assumed that people with less self-regulative skills are more vulnerable to develop an IUD, also due to reduced cognitive control. Stodt et al. [22] demonstrated that higher technical and productive skills increase the risk to suffer from IUD symptoms, whereas higher reflective and self-regulative skills were negatively correlated with IUD symptoms within a German sample. Therefore, the latter domains of an Internet literacy can be seen as preventive factors, which are not stable but learnable. Nevertheless, the preventive values of specific Internet literacy domains and their mediating or moderating role in the addiction process is an important topic of research. In another German study, Wegmann et al. [21] showed that both Internet-use expectancies and self-regulation as one domain of Internet literacy mediate the effect between psychopathological predispositions and ICD symptoms and especially productive and self-regulative skills are correlated with expectancies towards the Internet. More detailed, higher productive and interactive skills as well as lower self-regulation abilities go along with higher expectancies regarding positive reinforcement and the avoidance of negative feelings. 

### 1.5. Aims and Hypotheses

Recent German studies declared IUD as a nationwide problem, e.g., [9]. Its symptoms are correlated with lower extraversion and higher neuroticism as well as the expectancies someone has towards using the Internet, and certain Internet literacy dimensions [20,21,22,44,57]. Besides this, most epidemiological studies revealed high prevalence rates in China and adjacent nations, e.g., [6,10]. Addressing personality, also higher neuroticism and lower extraversion are mostly reported as significant predictors of an IUD in China, even if some studies indicated correlations with all traits of the Big Five [28]. Regarding Internet literacy, just a few findings have been reported for Chinese samples [51], while from our knowledge no research has been done on Internet-related specific cognitions.

The first aim of our study was to investigate whether previous findings regarding the relationship of personality, Internet-use expectancies, different Internet literacy domains and IUD symptoms could be replicated across different nations and cultures. We expect that (i) primarily lower extraversion and higher neuroticism should be linked to higher tendencies towards IUD, (ii) higher Internet use expectancies should be accompanied with IUD symptoms and (iii) primarily higher productive and interactive as well as lower self-regulative skills should be correlated with higher tendencies towards an IUD. Secondly, we wanted to have a closer look at potential differences and interaction effects between the above-mentioned factors among two countries which differ in their cultural background and most likely in their Internet-use behavior.

## 2. Materials and Methods 

### 2.1. Participants

Overall, 821 participants (334 female, 487 male) from Germany and China took part in a multi-parted data acquisition. The mean age of the whole sample was *M* = 20.71 years (*SD* = 3.01) with a range from 16 to 30.

The German convenience subsample consisted of 411 participants (179 female, 232 male; age: *M* = 20.70, *SD* = 3.34, range: 16–29 years). German participants were recruited from all over Germany by advertisements in university, e-mail lists, announcements on social networking sites, as well as word-of-mouth recommendation. Most of the German participants were university students (33.5%), of which 72.3% named the Abitur (German’s secondary school leaving diploma) and 13.1% the Fachabitur (vocational baccalaureate diploma) to be their highest school educational level. Additionally, 10.2% of the university students already received their bachelor’s degree and 2.2% received their master’s degree or a comparable graduation. Further, 25.2% of the German sample were pupils, 22.2% trainees, 10.3% were employees (of which 50.1% had a university degree), and 8.8% of the subsample practiced other professions. 

The Chinese convenience subsample consisted of 410 participants (155 female, 255 male; age: *M* = 20.72, *SD* = 2.65, range: 16–30 years). Chinese participants were recruited via announcements and postings in university as well as advertisements in university online forums. Overall, 202 Chinese participants took part in a data collection carried out in Beijing. Further 151 participants took part in the *Chengdu Gene Brain Behavior Project (CGBBP)* and have been recruited in Chengdu. An additional subsample of 57 participants took part in another data acquisition in Chengdu. The data collection was performed in these two Chinese cities due to location-dependent reasons (e.g., the studies were carried out in laboratories in these cities’ universities). Within all surveys, most of the Chinese participants were undergraduate students (74.6%), followed by graduate students (23.4%) and high school graduates (2.0%). The corresponding local ethics committees approved the study.

### 2.2. Measures 

Online surveys were used to gather participants’ information of the below mentioned measurements in Germany and China. The short Internet Addiction Test, the Internet-use Expectancies Scale, and the Internet Literacy Questionnaire were already available in German language and professionally translated into Chinese. Due to methodological issues, which are described later, two different personality questionnaires were used in the two countries. In the German subsample, the 10-Item Big Five Inventory (BFI-10) was used to assess the Big Five personality traits, whereas the NEO Five-Factor Inventory was used in the Chinese subsample. Both questionnaires were already available in the respective languages. In former studies, all of the used questionnaires showed good psychometric properties and validity. In this study’s sample, all questionnaires and its subscales revealed a good internal consistency with Cronbach’s *α* > 0.700, except for the subscale *openness* and *agreeableness* of the Chinese NEO Five-Factor Inventory, which however were still in the acceptable range. All reliabilities (Cronbach’s *α*) of the used questionnaires are presented in Table 1. Cronbach’s *α* reliabilities of the BFI-10 are not reported because this measure is not appropriate for calculating Cronbach’s *α* as only two items are building each scale [58]. 

We further compared the factorial structure of the short Internet Addiction Test, the Internet-use Expectancies Scale, and Internet Literacy Questionnaire using mean structure analyses with nation as grouping variable. Here, the model fits revealed no good fit with the data indicating that the factorial structure of the scales seem to be different between the nations. Nevertheless, all items scored significantly on their respective factor/latent dimension with acceptable to good factor loadings in both nations (weakest factor loading = 0.350, highest factor loading = 0.896). This issue will be further discussed in the limitations section.

#### 2.2.1. Short Internet Addiction Test (s-IAT) 

To measure symptoms of unspecified IUD, the short Internet Addiction Test (s-IAT; [19]) was used. It is a 12-item short version of Young’s original Internet Addiction Test (IAT; [59]), an internationally esteemed questionnaire to cover IUD symptoms. The s-IAT has already been validated and frequently used in past research. Within this questionnaire, participants have to evaluate their subjective complaints and experienced negative consequences due to excessive online activities in everyday life. Besides the total sum score, the questionnaire consists of the two subscales *loss of control/time management* and *craving/social problems*, each consisting of six items. Items were rated on a 5-point Likert scale from 1 (*never*) to 5 (*very often*). The total sum score ranges from 12 to 60, in which a higher sum score of the s-IAT indicates higher symptoms of an IUD. To evaluate the strength of IUD symptoms, the cut-off for the total score was >30 to indicate a problematic Internet use and >37 to indicate a pathological Internet use [19]. The questionnaire in English, German, and Chinese language is reported in Table A1.

#### 2.2.2. Big Five Inventory-10 and NEO Five-Factor Inventory

Because of methodological issues, we used two different questionnaires to measure the Big Five personality traits. These two questionnaires represent reliable and valid instruments for the measurement of the above-mentioned traits in the respective country. For the German data collection, we preferred to use the Big Five Inventory-10 (BFI-10; [60]), which (despite its shortness) represents a well-evaluated and valid instrument in Western countries. While the validity of the BFI-10 in Chinese language still has to be further tested [61], we decided to use the 60-item version of the NEO Five-Factor Inventory (NEO-FFI; [62]), which has been used in several Asian studies before and revealed a good validity, e.g., [63]. Both questionnaires were used to measure the participants’ level of *neuroticism*, *extraversion*, *openness*, *conscientiousness*, and *agreeableness*. In both questionnaires, higher mean scores reveal a higher level of the respective characteristic.

The BFI-10 consists of 10 items with two items on each trait. All of the items were rated on a 5-point Likert scale ranging from 1 (*strongly disagree*) to 5 (*strongly agree*).

The NEO-FFI comprises overall 60 statements, which have to be evaluated on a 5-point Likert scale from 1 (*strongly disagree*) to 5 (*strongly agree*). 

#### 2.2.3. Internet-Use Expectancies Scale

To measure expectancies and motivating factors for using the Internet in general, the Internet-use Expectancies Scale (IUES; [44]) was used. The scale consists of the two dimensions *positive reinforcement* and *avoidance expectancies* (each having four items). The dimension positive expectancies measures positive feelings, which are accompanied by using the Internet, whereas the dimension avoidance expectancies indicated whether the Internet is used to avoid negative feelings. Every item was answered on a 6-point Likert scale with a range from 1 (*completely disagree*) to 6 (*completely agree*). Mean scores for each subscale have been calculated. Although the IUES is a relatively new developed questionnaire, it has already been used in several studies [20,21,44] and has also been adapted for specific Internet-use patterns, like Internet-communication, e.g., [57]. The questionnaire in English, German, and Chinese language is shown in Table A2.

#### 2.2.4. Internet Literacy Questionnaire

The Internet Literacy Questionnaire (ILQ; [22]) was applied to measure the individuals’ competent and adequate dealing with the Internet on the following four dimensions: *technical expertise*, *reflection and critical analysis*, *production and interaction*, and *self-regulation*. Depending on a new calculated exploratory factor analysis, a more economical version of the ILQ was used. The shortened version includes 18 items on the four above-mentioned dimensions, compared to the original 24-item version. According to Stodt et al. [22], the first dimension *technical expertise* measures “the individual’s expertise in handling computer hard- and software as well as Internet applications” ([22], p. 31) on four items. The dimension *production and interaction* covers five items measuring “how and why an individual uses the Internet to create own content and to interact with others” ([22], p. 31). The third dimension *reflection and critical analysis* covers “the individual’s ability to evaluate the credibility of online content and the behavior of others as well as critically reflecting one’s own activities on the Internet” ([22], p. 31) on four items. The dimension *self-regulation* consists of five items covering “the individuals’ ability to regulate their own Internet use to prevent negative consequences for daily life” ([22], p. 31). Every item had to be answered on a 6-point Likert scale ranging from 0 (*strongly disagree*) to 5 (*strongly agree*). Mean scores were calculated for each dimension. 

Due to translation problems, item number 8 from the Chinese version of the ILQ had to be removed. Therefore, the subscale *reflection and critical analysis* consists of 3 items in the Chinese version. The subscales’ internal consistency was still in a good range (Cronbach’s *α* = 0.723). Nevertheless, we provide readers with the complete questionnaire in English, German, and Chinese language as presented in Table A3, including a revised version of item number 8. 

### 2.3. Statistical Analysis Section 

Aside from the presentation of descriptive statistics, inferential statistics are provided in the following result section with respect to both the German and Chinese sample. *t*-Tests were used to examine differences between both nations regarding the manifold Internet variables, whereas Pearson’s correlations were used to provide insights into correlation patterns between Internet variables in both countries. In addition, moderated regression analyses were used to consider possible interaction effects between nationality and Internet literacy within the development and maintenance of an IUD. The statistical analyses were performed by using IBM SPSS 24. MPlus version 6.12 (IBM, Armonk, NY, USA) was used to carry out the mean structure analyses. Reported effect sizes are in accordance with Cohen’s *d* (small effect: *d* = 0.30; medium effect: *d* = 0.50; large effect: *d* = 0.80) for *t*-tests and *r* (small effect: *r* = 0.10; medium effect: *r* = 0.30; large effect: *r* = 0.50) for Pearson correlations [64]. 

## 3. Results

### 3.1. Descriptive Results

Table 2 contains the whole sample’s (German and Chinese collapsed) descriptive statistics of the different questionnaires including average scores, standard deviations, and range.

#### 3.1.1. Occurrence of IUD

**Figure 1:**
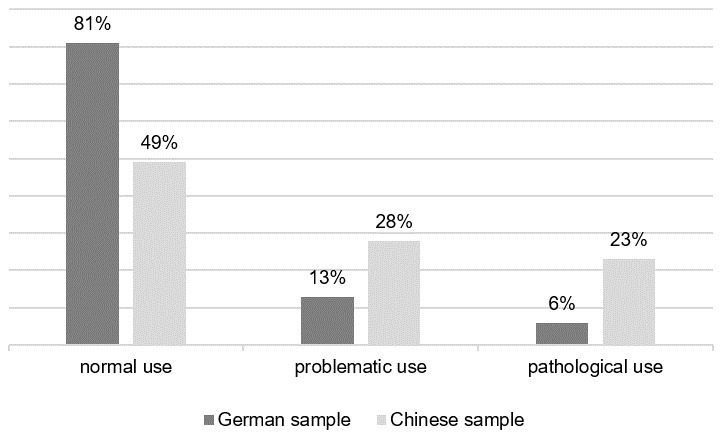
Internet-use disorder symptoms in the German and Chinese sample.

Based on the suggested cut-off scores of the s-IAT by Pawlikowski et al. [19], 55 participants of the German sample indicated a problematic use and 25 participants reported symptoms of a pathological use of the Internet. In China, 115 of the participants reported a problematic, 95 a pathological use. Figure 1 illustrates the percentage distribution of IUD tendencies in both countries.

### 3.2. Inferential Statistical Analyses

#### 3.2.1. Testing Both the Chinese and German Sample for Differences in Age and Gender Ratios

Both samples did not differ with respect to age (*t* = 0.12, *p* = 0.901) and gender ratios (*χ*^2^ = 2.81, df = 1, *p* = 0.094). However, since both subsamples were tried to be parallelized as good as possible regarding sample size and age, no age differences were expected. In terms of gender ratios, both samples show merely slight descriptive differences. We further tested for significant age and gender differences in tendencies towards IUD, Internet-use expectancies, Internet literacy, and personality in both samples. Here, age was significantly associated with IUD in the Chinese (*r* = 0.142, *p* = 0.004), but not in the German sample (*r* = −0.072, *p* = 0.145). Regarding gender effects, male participants in the German sample indicated higher scores in the s-IAT compared with females (*t* = 1.98, *p* = 0.049; *M* = 24.81, *SD* = 7.48 versus *M* = 23.35, *SD* = 7.38). No significant gender differences in the Chinese sample could be observed (*t* = 0.46, *p* = 0.644; *M* = 31.53, *SD* = 8.89 versus *M* = 31.08, *SD* = 10.30). While investigating the hypothesized cultural differences, we additionally controlled for the variables of age and gender. Calculated ANCOVAs revealed that the significant cultural effects on the observed variables reported below are not caused by age and gender effects. Nevertheless, some significant effects of age and gender on the measured Internet and personality variables in both countries could be observed. For an overview please see Tables S1 and S2 in the Supplementary Materials.

#### 3.2.2. Comparative Analyses

In a first step, *t*-tests for independent samples including effect sizes by Cohen [64] were calculated to compare the different scores of the s-IAT, ILQ, IUES, and the personality questionnaires of both countries. The analyses revealed significant differences between Germany and China regarding the s-IAT total score and both subscores with medium to high effect sizes, in which the Chinese sample indicated higher scores. Concerning Internet-use expectancies, also the Chinese sample showed higher scores regarding the *positive reinforcement* and *avoidance expectancies* with medium to high effect sizes. Regarding the self-evaluated Internet literacy, Chinese participants indicated significant higher scores in the dimension *production and interaction* (medium effect size), whereas the German sample scored higher on the dimension *reflection and critical analysis* (small effect size). No significant differences were found regarding the dimensions *technical expertise* and *self-regulation*. With regard to personality differences, the Chinese sample reported significantly higher scores of *neuroticism* and *agreeableness* than the German sample (small effect size). For *extraversion* and *openness*, German participants indicated higher scores (small to medium effect sizes). No significant differences were found regarding the trait *conscientiousness* (see Table 3).

#### 3.2.3. Correlations

Table 4 shows bivariate correlations between the s-IAT and the Big Five personality traits, divided by country. In the German sample, *neuroticism* (positive), *extraversion*, and *conscientiousness* (both negative) indicated a significant relationship to the s-IAT with low effects. For the Chinese sample, all personality traits of the Big Five showed significant relations with the s-IAT. *Neuroticism*, *conscientiousness*, and *agreeableness* indicated the highest effect sizes. Except for *neuroticism*, all scores of the Big Five were negatively related to the scores of the s-IAT. According to Fisher’s *z* comparisons, the correlations between *neuroticism*, *extraversion*, *conscientiousness*, *agreeableness*, and the s-IAT were significantly different between both countries (see Table 4).

Table 5 includes the bivariate correlations between the s-IAT, ILQ and IUES scores, divided by country. For both countries, Internet-use expectancies correlated positively with the s-IAT total score and both subscores with medium to high effect sizes. The ILQ dimension *technical expertise* showed small significant correlations with the s-IAT scores in both samples. Here, the correlation between *technical expertise* and the s-IAT subscore *craving/social problems* differs significantly between Germany and China. The ILQ dimension *production and interaction* correlated positively with symptoms of an IUD in both countries. Regarding the domain *reflection and critical analysis*, the correlations just revealed significant positive and small effects with the s-IAT total score and the subscore *craving/social problems* in the Chinese sample. Here, Fisher’s *z* revealed significant differences between the countries. Lastly, the ILQ score for *self-regulation* showed significant negative correlations with IUD symptoms in the German but not in the Chinese sample (except for one s-IAT subdimension), resulting in a significant Fisher’s *z* comparison.

#### 3.2.4. Analyses of Interaction Effects

Based on the relationships found between certain Internet literacy domains and IUD symptoms as well as the different correlation effects between both samples, we calculated further moderated regression analyses to consider possible interaction effects between Internet literacy and the participants’ cultural background explaining the level of IUD symptoms. Because particularly the two domains *reflection and critical analysis* as well as *self-regulation* revealed high cross-cultural differences regarding their association with IUD symptoms, we used these two domains as the predictors in the upcoming analyses. 

In the first analysis, the ILQ score for *reflection and critical analysis* explained no significant amount of variance in the s-IAT total score (*R*^2^ = 0.001, *F*(1, 819) = 0.83, *p* = 0.361). Nevertheless, the country (∆*R*^2^ = 0.155, ∆*F*(1, 818) = 149.94, *p* < 0.001) and the interaction term of both variables explained a significant part of variance (∆*R*^2^ = 0.012, ∆*F*(1, 817) = 11.75, *p* = 0.001). The whole moderation model explained 16.8% of the s-IAT score’s variance (*R*^2^ = 0.168, *F*(3, 817) = 54.89, *p* < 0.001; see Table 6, model 1 for beta-coefficients).

**Figure 2:**
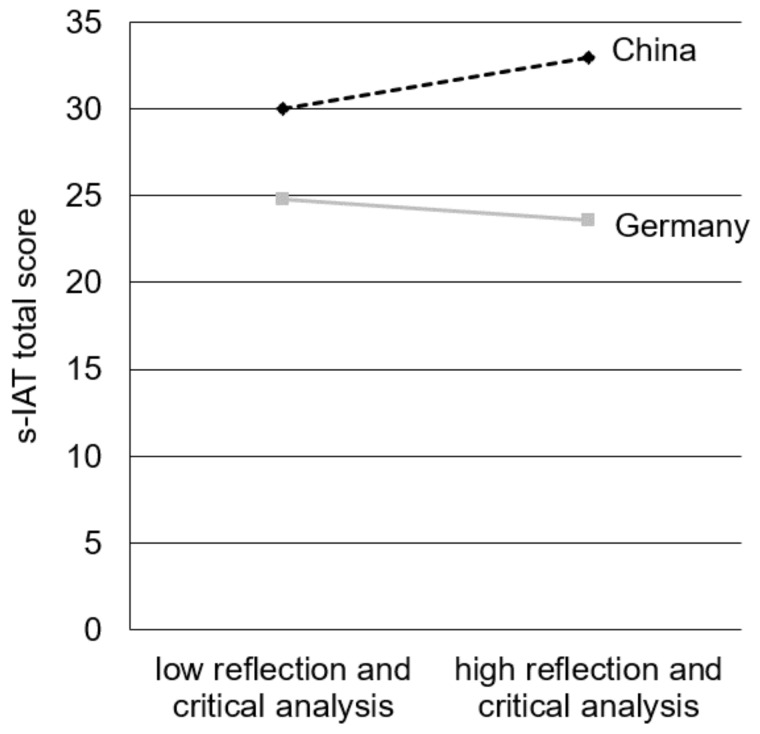
Simple slopes to illustrate the interaction effect between reflection and critical analysis and country on the s-IAT total score.

For further illustration of this interaction effect, we calculated simple slope analysis (see Figure 2). The slope illustrating the Chinese sample was significantly different from zero (*t* = 3.73, *p* < 0.001). The slope for Germany was not significant (*t* = 1.32, *p* = 0.187). These results indicate that there was an effect of reflective skills on the level of IUD symptoms in the Chinese sample. More detailed, Chinese participants with higher reflective skills tend to indicate higher s-IAT total scores in contrast to participants with lower skills. In the German sample the domain of *reflection and critical analysis* revealed no significant effect on IUD symptoms. 

Secondly, we calculated a moderated regression analysis where we added the ILQ domain *self-regulation* as the predictor. Here, the score for *self-regulation* (*R*^2^ = 0.078, *F*(1, 819) = 68.83, *p* < 0.001), the country (∆*R*^2^ = 0.140, ∆*F*(1, 818) = 145.76, *p* < 0.001), and the interaction term of both variables explained a significant part of variance (∆*R*^2^ = 0.026, ∆*F*(1, 817) = 27.90, *p* < 0.001). All variables explained 24.3% of the s-IAT score’s variance (*R*^2^ = 0.243, *F*(3, 817) = 87.37, *p* < 0.001; see Table 6, model 2 for beta-coefficients).

**Figure 3:**
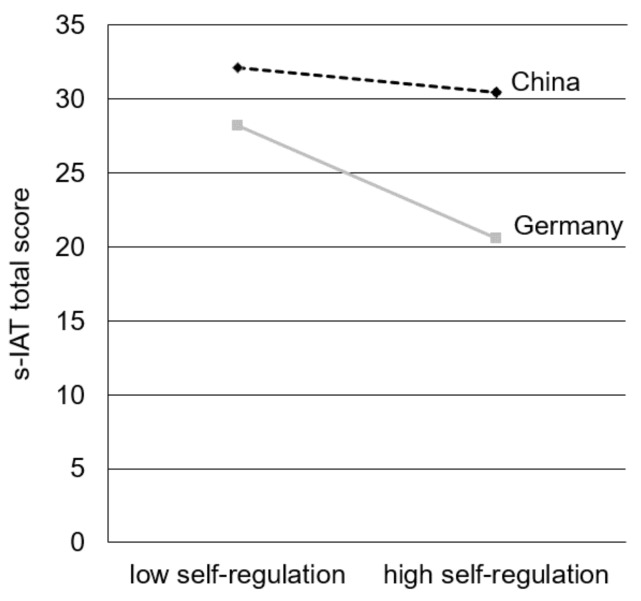
Simple slopes to illustrate the interaction effect between self-regulation and country on the s-IAT total score.

Simple slope analysis revealed significant slopes for the Chinese (*t* = 2.08, *p* = 0.038) and the German sample (*t* = 9.69, *p* < 0.001). However, while the effect of self-regulating skills is relatively low in the Chinese sample, in the German sample higher self-regulative skills reduce the score for IUD symptoms considerably in contrast to the Chinese sample (see Figure 3). 

## 4. Discussion

This study is the first one investigating a cultural comparison of the relationship between personal core characteristics, specific Internet-related cognitions and skills, as well as symptoms of an IUD. The present study’s first aim was to verify whether previous findings on the effect of personality traits as well as state variables like Internet-use expectancies and Internet literacy on the development and maintenance of an IUD are stable across different cultures and nations. Another purpose was to point out potential differences in the relationship of these variables between two countries from different cultural areas. 

### 4.1. IUD Symptoms

By using the same questionnaire across both countries, we found significant higher IUD symptoms in the Chinese sample, where the average total score of the s-IAT was almost at the cut-off score for a problematic Internet use. The significant cultural difference is reflected by the prevalence rates found in both countries: Whereas 6% of the German sample indicated a pathological Internet use, which value is in line with former epidemiological studies [7,65], 23% of the Chinese sample indicated an excessive and pathological use of the Internet. This rate is much higher than the prevalence reported for China in recent studies, where the rates range between 8 and 15 percent [66,67,68]. A different age range as well as different locations of recruitment in this study may explain the higher prevalence compared to previous investigations. Further, the year of publication could explain varying IUD frequencies, since prevalence rates could have been grown in the recent five years. Higher prevalence rates of IUD in Asian countries with respect to Western cultures have also been reported in the past [7,14]. However, because of the missing clinical evidence and differentiating diagnostic criteria of the methods used, reported prevalence rates generally have to be compared with caution.

### 4.2. Theoretical Integration of the Results

In the following, we want to integrate the results of this study into a recently published theoretical framework, called the I-PACE model (I-PACE stands for Interaction of Person-Affect-Cognition-Execution). In this empirically based model, Brand et al. [2] illustrate different stages of the addiction process of specific Internet-use disorders. The model illustrates the interaction of person’s core characteristics, such as personality traits, social cognitions, and psychopathological symptoms (P-component) and possible mediating or moderating state variables like coping styles, Internet-related cognitive biases, as well as affective and cognitive responses (A/C-component). According to the authors, the interaction of these variables lead to the decision to use a certain application (E-component). The later experienced gratification could positively reinforce such mediating and moderating effects, resulting in an addictive use. 

#### 4.2.1. Personality

In accordance with the addiction process described in the I-PACE model, we start to discuss our findings regarding the Big Five personality traits. The found cultural differences in the traits extraversion, openness, and neuroticism are in line with a worldwide comparative study, where East Asian countries indicated the lowest scores of extraversion and openness as well as the highest scores in neuroticism [37]. The results on conscientiousness (no significant difference) and agreeableness (higher scores in China), are in line with typical national stereotypes. Both German and Chinese citizens are described as working hard to reach their goals and having a strong will power [69,70,71,72]. This cultural commonality might be reflected by a comparable score in the conscientiousness scale of both samples. In addition, China is considered as a nation tending to hierarchical structures and following social norms [69]. This could be reflected by higher levels of agreeableness of the Chinese sample compared to the German sample. However, the agreeableness finding speaks against observations, where Chinese samples scored significantly lower on agreeableness compared to a German sample [63,73]. It remains difficult to interpret and ascribe the found personality differences solely to cultural differences. Therefore, the results of this study have to be interpreted cautiously. Previous German and Chinese studies on the association between personality, general Internet use and IUDs revealed controversial results, but most consistent relations have been found between IUDs, low conscientiousness and high neuroticism [2,19,74,75,76]. Following this, we could show similar findings by indicating significant correlations between high neuroticism, low conscientiousness and IUD symptoms in both countries. Both traits revealed higher effect sizes in the Chinese sample, meaning that higher levels of neuroticism and lower levels of conscientiousness are more strongly associated with IUD symptoms in China than in Germany. Our results also demonstrate negative correlations between openness, agreeableness, and IUD symptoms in the Chinese sample. Regarding extraversion, significant relationships in both countries were found, which do not significantly differ. While interpreting these results, it must be considered that some personality factors have not been extracted consistently over several cultural regions [37]. Furthermore, the Big Five personality model mostly displays typical traits of people living in Western individualistic cultures but not of citizens in Asian cultures, which are more collectivistic [37]. This could lead to the different effects of some of the Big Five personality traits on IUD symptoms in both samples.

#### 4.2.2. Internet-Related Cognitions and Skills

The next level of the I-PACE model comprises mediating and moderating state variables between the P-component and the decision to use a certain application. In this study, this component is represented by Internet-use expectancies and Internet literacy. Regarding Internet use expectancies, we found that Chinese participants indicated higher expectancies towards the Internet as a useful tool to reduce stress or to feel confident compared with the German sample. This is in line with Li and Kirkup [13], who found cultural differences between British and Chinese students regarding Internet experiences and attitudes. Although higher use expectancies were accompanied with higher IUD scores in both countries, no significant cultural differences regarding the strength of this relationship were found. There are already some empirical German studies underlining Internet-use expectancies as mediating variables, reinforcing the effect between personal predispositions and IUD symptoms [20,21,44,57]. Our study’s results suggest similar effects in China, but further research with mean structure analyses is needed to investigate the role of Internet-use expectancies in the addiction process in a cross-cultural comparison. 

Even though Internet literacy covers no inherent part in the I-PACE model, previous studies already indicated that Internet-related skills play an important role in the addiction process, e.g., [21,22,51]. Further, the dimensions of Internet literacy postulated by Stodt et al. [22] share similarities with Internet-related cognitive biases, as productive and interactive skills are a result of the subjective awareness of today’s technological possibilities. In addition, the possession of self-regulative skills is theoretically accompanied with addiction-related concepts like cue-reactivity and craving as well as coping mechanisms. This study indicates less productive and interactive skills as well as higher reflective and analyzing skills of the German compared to the Chinese sample. Besides that, our analyses revealed two further interesting findings on the interaction between Internet literacy and IUD symptoms across both countries. Firstly, reflective and analytical skills seem to play no role in the development and maintenance of an IUD in German individuals. In contrast, we found a positive relationship between reflective skills and IUD symptoms in the Chinese sample. Simple slope analyses illustrated that Chinese participants show higher symptoms of an IUD compared to the German sample, but surprisingly indicate the highest symptomatology, when reflective skills are well-marked. At first site, this result appears to be counterintuitive, since reflective skills have been emphasized as preventive factors in past studies, e.g., [22]. However, it can be assumed that culture affects in what way an effect of reflective skills on IUD symptoms can occur. People living in collectivistic cultures like China are described as being more concerned about the effect of one’s own behavior on others [11]. It can be assumed that the possession of reflective skills is necessary to evaluate this effect. In fact that in online environments such as games or social networks the interaction with others displays an essential feature, it could be that especially collectivistic thinking people with high reflective skills tend to use such applications more frequently and intense while neglecting personal needs and duties, leading to higher IUD symptoms. Past studies already investigated that people living in collectivistic countries are associated with a preference for online social interaction and appreciate their online social network [77,78].

Secondly, the ability to self-regulate one’s own Internet use had just a low negative effect on the level of IUD symptoms among Chinese participants. On the other hand, the effect of self-regulation was much higher in the German sample, where individuals who cannot self-regulate their Internet-use behavior showed higher IUD symptoms compared to the people who have good self-regulation skills. The latter finding is in line with former research, where people with symptoms of an IUD reported problems in regulating and controlling their own Internet-use [21,22]. Regarding Cloninger [79], higher self-regulation abilities (in the offline context) are associated with higher self-directedness. Furthermore, people scoring high on self-directedness indicate high levels of self-esteem, will-power, and are self-satisfied with their own personality and problem-solving. As a result the authors emphasized self-directedness as an important personality dimension affecting personality disorders. Further, less self-directed people indicate higher symptoms of an IUD [29,30,80,81,82,83]. In a cross-cultural study by Sariyska et al. [30], however, the relation between self-directedness and IUD was slightly different across countries. Here, the effect sizes in two Chinese samples were lower than in the German sample. Our study found that even though online-focused self-regulation is not different across both investigated countries, it has no significant impact on IUD in China. Putting it together with previous research, these findings suggest the existence of heterogeneous mechanisms and effects regarding the interaction of online-focused self-regulation skills, self-directedness, and IUD symptoms in different countries and cultures.

### 4.3. Further Theoretical Integration

In the following, further reasons regarding the described cultural differences are discussed. The continuum of collectivism and individualism represents a frequently studied dimension of cultural variability, showing high differences such as between European and Asian countries, e.g., [12,84,85]. While European population indicates a higher degree of individualism, resulting from being more concerned about consequences of one’s own behavior, needs, and goals, Asian population displays higher degrees of collectivism, represented by being more concerned of one’s own behavior on others, collective needs, and group goals [11,12]. Further, individuals living in collectivistic societies show greater levels of a need to belong, while members of individualistic cultures show a greater need for self-presentation [11,84,86]. Also comparing a German and a Chinese sample, a recent study by Montag et al. [12] pointed out that low acceptance of power distance as a predictive factor for IUD symptoms, especially in males and Chinese individuals. The authors assume that an individual’s greater acceptance of power distance and the higher exerted power on themselves leads to the desire to find a way and place to relieve from stress, which could be the Internet.

Furthermore, China displays the world’s fastest growing Internet population [87] with an increasing online gaming industry [88]. Especially in today’s social, mobile, or online role-playing games, it is necessary for users to play together with others to reach certain group goals. It can be assumed that the collectivistic orientation within the Chinese society contribute to a high involvement in online gaming as well as social networking due to its collaborative features. Further, the competitive character of online games as well as further social factors (e.g., socializing, giving support, teamwork) display important motivational factors for playing online games [89,90], which could be more distinct in collectivistic cultures. Specific using motives have been assumed as significant predictors of an addictive behavior [2]. Further studies should examine potential effects of collectivism/individualism in different countries and its impact on (excessive) Internet use.

### 4.4. Practical Implications for Both Countries

Although both investigated samples are not representative for its respective population, we think that this study’s results could give a contribution to prevention and intervention programs in both nations, which are mentioned in the following.

#### 4.4.1. Practical Implications for Germany

The results of the current study point IUD out as a significant problem within the investigated German sample, where especially two Internet literacy domains are related to higher symptoms: higher productive/interactive as well as lower self-regulative skills. Besides, no considerable effect of technical and reflective skills on tendencies towards an IUD have been found. These correlations strengthen the findings by Stodt et al. [22], who stated that pure technical skills do not counteract a dysfunctional Internet use. As demonstrated in the I-PACE model and relying on empirical investigations, Internet-related cognitions and skills play a key role in the process of developing a specific Internet-use disorder [2,20,21]. Such state factors could be addressed in specific training programs and probably diminish the effect of dysfunctional personality factors and psychopathological symptoms on the development of an IUD. Currently, the improvement of self-regulative skills (without disregarding technical knowledge) still does not find its way into today’s curricula of media and/or Internet literacy in Germany. Based on the current findings, we recommend to include the improvement of self-regulative skills in current curricula of media competence in Germany.

#### 4.4.2. Practical Implications for China

Statistics revealed high occurrence of a pathological use of the Internet as well as a high number of people who indicated a problematic use in the investigated sample, mostly consisting of university students. Taking the pathological and the problematic groups together, more than half of the Chinese sample indicated at least a problematic Internet use, pointing towards a serious health problem in these Chinese students. Previous studies from China showed that especially university freshmen suffer from higher IUD symptoms, often associated with psychopathological symptoms, such as depressive symptoms, social anxiety, or higher levels of loneliness [24]. These findings emphasize the importance of prevention and intervention programs not only in middle school, but also in university. Such programs should screen for high-risk Internet users and focus on the improvement of Internet-related cognitions and skills that are related to IUD symptoms, as the current study shows. More detailed, Chinese participants indicated to have high expectancies towards the use of the Internet. Those people who think that the Internet is helpful to avoid negative feelings, like stress and loneliness, or to gain positive emotions to a high degree tend to use the Internet more excessively. That is why prevention and intervention programs in China should focus on the outcomes people expect by using certain Internet applications and should demonstrate alternative coping strategies. Besides that, self-regulation skills are not correlated with the s-IAT total score in the Chinese students, which is different from the German sample. Nevertheless, based on the results of the current study that specific skills play a significant role in the development and maintenance of an IUD, we propose to include such skills in current prevention programs. Future studies should address how these skills could be systematically integrated in upcoming prevention programs.

### 4.5. Limitations

Some limitations of this study need to be addressed in the following. First, the use of two different questionnaires for measuring the Big Five personality traits, the BFI-10, which was used in the German sample, and the NEO-FFI, which was used in the Chinese sample, could be a reason for the different results regarding the personality traits and their impact on IUD symptoms in both samples. Nevertheless, the use of these two questionnaires was carefully considered during planning the study. In previous studies, the BFI-10 displayed a valid and reliable instrument with good psychometric properties in German samples. Further, its 10-item version is very efficient and advantageous for the use in online studies, where time is limited. For the best of our knowledge, there is no valid and evaluated short version of a Big Five questionnaire in Chinese language (to be more precise: please see that the BFI-10 has been published in Mandarin, recently, but a problem concerning one introversion/extraversion item is discussed in Lachmann et al. [61]). That is why we decided to use the 60-item version of the NEO-FFI, which has been used in several Asian studies before and therefore displays a well-evaluated instrument. Although, the NEO-FFI is also evaluated sufficiently in Germany and other Western countries, we used the BFI-10 in the German data collection due to practical reasons and its above mentioned advantages. 

One further limitation is the removal of one item out of the subscale *reflection and critical analysis* of the ILQ in the Chinese sample. We had to remove this item directly after data collection because of a problematic translation, which could lead to different possible understandings by the participants. Additionally, the removal should ensure the subscale’s content validity. Regarding the subscale’s internal consistency, Cronbach’s α was still in the acceptable range. With the removal of one item, we do not expect different results because the remaining items substantially represent the same theoretical facet. For the German sample, we additionally calculated all analyses including the subscale *reflection and critical analysis* again with the same three items as used in the Chinese sample. The results did not differ between the three- and four-item solutions.

As a further limitation, the different factorial structures of the s-IAT, IUES, and ILQ in both nations, which are based on weak model fits in mean structure analyses, have to be mentioned. Even if the analyses revealed non-acceptable model fits, we had a closer look on the factor loadings within both nations. Here, every item loaded significantly on its theoretically suggested and empirically approved factor with just slightly varying effects between both nations. Therefore, we assume that the results of the current study are quite comparable. Further, former studies already demonstrated the applicability of the s-IAT and the IUES in a cross-cultural setting, e.g., [57]. However, further studies are needed to test if these questionnaires are suitable for investigating cross-cultural issues.

Furthermore, limitations regarding the representativeness of both investigated samples exist. First, in China and Germany data was collected from convenience samples. Whereas German participants came from all over Germany and were not recruited in a specific region, Chinese data was collected in two certain cities exclusively due to location-dependent reasons, which may have led to region-based effects. Second, the Chinese sample almost consisted of university students, whereas the German sample also contains participants who are employed. Strictly speaking, these facts reduce the level of generalizability and interpretation of the findings in terms of underlying cultural differences. Nevertheless, the German sample was predominately well educated with a high number of participants holding a university degree. Furthermore, both samples did not differ in age and gender. At this point, we want to clarify that this study did not have the intention to examine cultural differences with two nationally representative samples. Notwithstanding, although several limitations exist that have to be beared in mind, this study indicated several similarities between both nations (e.g., level of Internet-use expectancies and personality correlations) which are theoretically grounded and indicate global effects. On the other hand, the differences found between both nations (influence of reflective and regulative skills) have to be interpreted with caution. Here, further replications are necessary to check the meaningfulness of these results. Hence, this study’s findings provide evidence that cultural differences between Germany and China regarding symptoms of an IUD and influencing trait and state variables could exist. However, these differences need to be replicated and further investigated in upcoming research explicitly. 

### 4.6. Future Directions

This study suggests some directions for upcoming research. Future studies should address possible mediating and moderating effects between personality traits, Internet-related cognitions and skills, as well as IUD symptoms across different countries such as by using structural equation modelling and mean structure analyses. Based on the findings of this study, examining certain parts and interaction effects of the I-PACE model with representative samples from different countries would be helpful promoting a better understanding of cultural differences in IUD. Additionally, further empirical studies should focus on the role of one person’s knowledge and skills, which could represent additional mediating or moderating factors in the addiction process but have not been a part of previous theoretical models. Furthermore, there are just a few longitudinal studies covering IUD. The investigation of long-term effects in different countries and cultures would be helpful to shed light onto the reinforcing or protective value of Internet literacy and further Internet-related cognitive biases on the development and maintenance of an IUD and respective cultural differences. Especially, longitudinal designs would be helpful to develop specific prevention and intervention programs.

## 5. Conclusions

The present study strengthens previous findings on the effect of personality traits as well as state variables such as Internet-use expectancies and Internet literacy domains on the development and maintenance of an IUD within two samples from different countries and cultural areas. In addition, some interesting country-specific differences regarding these variables and their relationship to symptoms of an IUD were pointed out. Next to a higher occurrence of IUD symptoms in the Chinese sample compared to the German sample, Chinese participants scored significantly higher on the Big Five personality traits of neuroticism and agreeableness, whereas German participants scored higher on the traits of extraversion and openness. However, please note that different inventories to measure the Big Five personality traits were applied in each country. Furthermore, Chinese individuals showed higher Internet-use expectancies to avoid negative feelings or to be positively reinforced. Further analyses indicated that Chinese participants with higher reflective skills regarding their Internet behavior indicated highest IUD symptoms, whereas such skills indicated no effect within the German sample. Moreover, higher skills in self-regulating one’s own Internet use were accompanied with lower IUD symptoms in the German, but not in the Chinese sample. Despite the missing representativeness of both samples and some limitations of the current study, several theoretically grounded findings have to be underlined, strengthening the global applicability of this study and providing evidence that cultural differences between Germany and China regarding IUD symptoms and influencing trait and state variables could exist. Because Internet literacy domains seem to play a significant role in the development and maintenance of an IUD, we propose to encourage skills such as reflective and self-regulating skills in current or future IUD prevention programs, which should be individually matched to each countries conditions. Upcoming research should address the replication of this study’s results to check its meaningfulness.

## Figures and Tables

**Table 1:** Overview on reliabilities (Cronbach’s *α*) of the NEO Five-Factor Inventory (NEO-FFI) *, Internet-use Expectancies Scale (IUES), Internet Literacy Questionnaire (ILQ), short Internet Addiction Test (s-IAT) and their subscales in the German and Chinese samples.

	Germany	China
NEO-FFI—*Neuroticism*	-	0.820
NEO-FFI—*Extraversion*	-	0.732
NEO-FFI—*Openness*	-	0.602
NEO-FFI—*Conscientiousness*	-	0.747
NEO-FFI—*Agreeableness*	-	0.698
IUES—*Positive reinforcement*	0.855	0.775
IUES—*Avoidance expectancies*	0.784	0.810
s-IAT—*Total score*	0.869	0.919
s-IAT—*Loss of control/time management*	0.819	0.862
s-IAT—*Craving/social problems*	0.809	0.870
ILQ—*Technical expertise*	0.805	0.775
ILQ—*Production and interaction*	0.759	0.834
ILQ—*Reflection and critical analysis*	0.707	0.723
ILQ—*Self-regulation*	0.772	0.810

* Note that in the German sample the BFI-10 was used, where no reliabilities can be presented.

**Table 2:** Descriptive statistics of the s-IAT, IUES, ILQ, BFI-10, and NEO-FFI.

Domain/Variable	*M*	*SD*	*Min–Max ^1^*
**Internet-use disorder (s-IAT)**			
Total score	27.76	9.23	12.00–60.00
Loss of control/time management	15.62	4.98	6.00–30.00
Craving/social problems	12.14	4.97	6.00–30.00
**Internet-use expectancies (IUES)**			
Positive reinforcement	4.00	1.11	1.00–6.00
Avoidance expectancies	2.93	1.23	1.00–6.00
**Internet literacy (ILQ)**			
Technical expertise	2.99	1.16	0.00–5.00
Production and interaction	2.52	1.72	0.00–5.00
Reflection and critical analysis	3.11	0.96	0.00–5.00
Self-regulation	3.00	1.00	0.00–5.00
**Big Five personality (BFI-10) ^2^**			
Neuroticism	2.66	0.95	1.00–5.00
Extraversion	3.53	0.96	1.00–5.00
Openness	3.46	1.08	1.00–5.00
Conscientiousness	3.30	0.90	1.00–5.00
Agreeableness	3.03	0.81	1.00–5.00
**Big Five personality (NEO-FFI) ^3^**			
Neuroticism	2.84	0.58	1.00–4.50
Extraversion	3.20	0.47	1.50–4.67
Openness	3.28	0.43	2.17–4.58
Conscientiousness	3.34	0.45	2.25–5.00
Agreeableness	3.38	0.46	2.00–4.83

^1^ Actual range of each scale, including the lowest and highest values measured in both subsamples. ^2^ Used in the German subsample (*n* = 411). ^2^ Used in the Chinese subsample (*n* = 410).

**Table 3:** Differences between the German and Chinese samples regarding the s-IAT, IUES, ILQ, and Big Five personality traits.

Domain/Variable	Germany		China		*t*	*df*	*p*	*d*
	*M*	*SD*	*M*	*SD*				
**Internet-use disorder (s-IAT)**								
Total score	24.17	7.46	31.36	9.44	−12.10	776.84	<0.001	0.85
Loss of control/time management	14.14	4.56	17.10	4.96	−8.89	819.00	<0.001	0.62
Craving/social problems	10.03	3.81	14.26	5.10	−13.47	757.47	<0.001	0.94
**Internet-use expectancies (IUES)**								
Positive reinforcement	3.70	1.20	4.31	0.92	−8.27	765.55	<0.001	0.57
Avoidance expectancies	2.47	1.14	3.40	1.13	−11.77	819.00	<0.001	0.82
**Internet literacy (ILQ)**								
Technical expertise	2.94	1.23	3.03	1.10	−1.06	808.87	0.291	0.08
Production and interaction	2.12	1.11	2.93	1.08	−10.59	819.00	<0.001	0.74
Reflection and critical analysis ^1^	3.18	0.88	3.03	1.09	2.29	798.88	0.022	0.15
Self-regulation	3.06	1.02	2.94	0.99	1.73	819.00	0.085	0.12
**Big Five personality ^2^**								
Neuroticism	2.66	0.95	2.84	0.58	−3.22	679.50	0.001	0.23
Extraversion	3.53	0.96	3.20	0.47	6.34	595.08	<0.001	0.44
Openness	3.46	1.08	3.28	0.43	3.10	536.97	0.002	0.22
Conscientiousness	3.30	0.90	3.34	0.45	−0.93	607.75	0.354	0.06
Agreeableness	3.03	0.81	3.38	0.46	7.57	650.46	<0.001	0.53

^1^ As already mentioned in the method section, the mean score of this dimension was calculated by using three instead of four items in the Chinese subsample. ^2^ German sample (*n* = 411): measured with BFI-10; Chinese sample (*n* = 410): measured with NEO-FFI.

**Table 4:** Correlations between s-IAT and Big Five personality traits ^1^ (Pearson correlations) including Fisher’s *z* comparison.

Domain/Variable ^2^	Germany			China			Fisher’s *z*		
	s-IAT Total Score	s-IAT LoC/TM ^3^	s-IAT C/SP ^3^	s-IAT Total Score	s-IAT LoC/TM ^3^	s-IAT C/SP ^3^	s-IAT Total Score	s-IAT LoC/TM ^3^	s-IAT C/SP ^3^
N	0.219 **	0.180 **	0.213 **	0.502 **	0.438 **	0.504 **	−4.70 **	−4.11 **	−4.83 **
E	−0.242 **	−0.183 **	−0.254 **	−0.186 **	−0.155 **	−0.195 **	−0.84	−0.41	−0.89
O	−0.012	−0.056	0.043	−0.160 **	−0.105 *	−0.193 **	2.13 *	0.70	3.40 **
C	−0.192 **	−0.204 **	−0.131 **	−0.365 **	−0.377 **	−0.308 **	2.69 **	2.71 **	2.66 **
A	−0.030	0.023	−0.085	−0.386 **	−0.275 **	−0.447 **	5.38 **	4.36 **	5.65 **

^1^ German sample (*n* = 411): measured with BFI-10; Chinese sample (*n* = 410): measured with NEO-FFI. ^2^ N = neuroticism, E = extraversion, O = openness, C = conscientiousness, A = agreeableness. ^3^ LoC/TM = Loss of control/time management, C/SP = Craving/social problems. * *p* ≤ 0.05, ** *p* ≤ 0.01.

**Table 5:** Correlations between s-IAT and Internet literacy as well as Internet-use expectancies (Pearson correlations) including Fisher’s *z* comparison.

Domain/Variable ^1^	Germany			China			Fisher’s *z*		
	s-IAT Total Score	s-IAT LoC/TM ^2^	s-IAT C/SP ^2^	s-IAT Total Score	s-IAT LoC/TM ^2^	s-IAT C/SP ^2^	s-IAT Total Score	s-IAT LoC/TM ^2^	s-IAT C/SP ^2^
**Internet-use expectancies**									
PR	0.390 **	0.352 **	0.344 **	0.369 **	0.338 **	0.356 **	0.35	0.23	−0.20
AE	0.567 **	0.507 **	0.505 **	0.556 **	0.480 **	0.564 **	0.23	0.51	−1.18
**Internet literacy**									
TE	0.105 *	0.120 *	0.062	0.153 *	0.069	0.215 **	−0.70	0.74	−2.23 *
PI	0.317 **	0.236 **	0.339 **	0.346 **	0.298 **	0.350 **	−0.47	−0.95	−0.18
RCA	−0.074	−0.064	−0.068	0.165 **	0.086	0.221 **	−3.44 **	−2.15 *	−4.18 **
SR	−0.516 **	−0.531 **	−0.375 **	−0.088	−0.185 **	0.018	−6.89 **	−5.77 **	−5.88 **

^1^ PR = positive reinforcement, AE = avoidance expectancies, TE = technical expertise, PI = production and interaction, RCA = reflection and critical analysis, SR = self-regulation. ^2^ LoC/TM = Loss of control/time management, C/SP = Craving/social problems. * *p* ≤ 0.05, ** *p* ≤ 0.01.

**Table 6:** Regression coefficients of the moderated regression analyses with the s-IAT total score as dependent variable.

	*B*	*SE (B)*	*β*	*t*	*p*
**Model 1**					
Reflection and critical analysis	0.44	0.31	0.046	1.42	0.156
Country	7.26	0.59	0.393	12.28	<0.001
Interaction	2.14	0.63	0.111	3.42	0.001
**Model 2**					
Self-regulation	−2.32	0.28	−0.252	−8.27	<0.001
Country	−6.91	0.56	−0.374	−12.278	<0.001
Interaction	−2.96	0.56	−0.161	−5.28	<0.001

## Supplementary Materials

The following are available online at http://www.mdpi.com/1660-4601/15/4/579/s1, Table S1: Gender differences in the s-IAT, IUES, ILQ, and Big Five personality traits in Germany and China (ANOVA), Table S2: Correlations between age and the observed Internet and personality (Pearson correlations) including Fisher’s *z* comparison between Germany and China.

## Appendix A

**Table A1:** Short Internet Addiction Test (s-IAT) in English, German, and Chinese language.

	English	German	Chinese
1.	How often do you find that you stay online longer than you intended?	Wie oft stellen Sie fest, dass Sie länger als beabsichtigt im Internet waren?	你经常会发现自己在网上呆的时间比你预期的要长么？
2.	How often do you neglect household chores to spend more time online?	Wie oft vernachlässigen Sie alltägliche Pflichten, um mehr Zeit online zu verbringen?	你经常会为了在网上多呆一会儿而忽略家务琐事么？
3.	How often do your grades or school work suffer because of the amount of time you spend online?	Wie häufig leidet Ihre schulische, Ihre universitäre oder Ihre berufliche Arbeit darunter, dass Sie so viel Zeit online verbringen?	你经常会因为上网用了很多时间而耽误了成绩或者大学的功课么？
4.	How often do you become defensive or secretive when anyone asks you what you do online?	Wie häufig reagieren Sie ausweichend oder verteidigend, wenn Sie jemand fragt, was Sie online tun?	你经常会在别人问起你在网上做什么的时候变得防御或者遮遮掩掩么？
5.	How often do you snap, yell, or act annoyed if someone bothers you while you are online?	Wie oft reagieren Sie patzig, schimpfen oder sind genervt, wenn Sie jemand stört, während Sie online sind?	你经常会因为别人打扰你上网而呵斥、大叫或者作出生气的行为么？
6.	How often do you lose sleep due to being online late at night?	Wie oft fehlt Ihnen Schlaf, weil Sie spät nachts noch online sind?	你经常会因为上网到半夜而失眠么？
7.	How often do you feel preoccupied with the Internet when offline, or fantasize about being online?	Wie oft denken Sie ans Internet, wenn Sie offline sind oder stellen sich vor, online zu sein?	你经常会在线下的时候总想着上网的事情或者想象着在网络上么？
8.	How often do you find yourself saying “just a few more minutes” when online?	Wie oft ertappen Sie sich dabei zu sagen: “Nur noch ein paar Minuten”, während Sie online sind?	你经常会发现在上网时你对自己说“再多几分钟”么？
9.	How often do you try to cut down the amount of time you spend online and fail?	Wie häufig versuchen Sie weniger Zeit im Internet zu verbringen und schaffen es nicht?	你经常试图减少上网的时间但是却以失败告终么？
10.	How often do you try to hide how long you’ve been online?	Wie häufig versuchen Sie zu verbergen, wie lange Sie online waren?	你经常会尝试隐瞒实际在网上呆的时间么？
11.	How often do you choose to spend more time online over going out with others?	Wie oft kommt es vor, dass Sie lieber mehr Zeit online verbringen als mit Anderen etwas zu unternehmen?	你经常会在网络上花费的时间比与他人一起外出的时间更多么？
12.	How often do you feel depressed, moody, or nervous when you are offline, which goes away once you are back online?	Wie oft fühlen Sie sich deprimiert, verstimmt oder nervös, wenn Sie offline sind – was sich ändert, wenn Sie wieder online sind?	你经常会因为下线而感到郁闷、不高兴或焦虑，而一旦上网这些感受就烟消云散了么？

Items must be answered on a 5-point Likert scale with the following response levels: 1 = never/nie/从不, 2 = rarely/selten/很少, 3 = sometimes/manchmal/有时, 4 = often/oft/经常, 5 = very often/sehr oft/非常常见. Scoring of the s-IAT scales: *Total score* is based on the sum score of all 12 items; *Loss of control/time management* is based on the sum score of the following items: 1, 2, 3, 6, 8, 9; *Craving/social problems* is based on the sum score of the following items: 4, 5, 7, 10, 11, 12.

**Table A2:** Internet-Use Expectancies Scale (IUES) in English, German, and Chinese language.

	English	German	Chinese
	I use the Internet because it makes possible/facilitates …	Ich nutze das Internet, weil es mir ermöglicht/erleichtert …	我使用网络是因为它在如下方面创造了可能性／便利性：
1.	to experience pleasure.	Freude zu erleben.	为了体验快乐。
2.	to distract from problems.	Problemen aus dem Weg zu gehen.	为了从问题（麻烦）中转移注意力。
3.	to have fun.	Spaß zu haben.	为了玩得愉快。
4.	to avoid loneliness.	Gefühle der Einsamkeit zu vermeiden.	为了避免孤独。
5.	to feel good.	mich gut zu fühlen.	为了感觉好。
6.	to escape from reality.	vor der Realität zu flüchten.	为了逃避现实。
7.	to gain positive emotions.	positive Gefühle zu erreichen.	为了获得正性情感。
8.	to avoid annoying duties.	lästige Aufgaben zu vermeiden.	为了回避恼人的责任。

Items must be answered on a 6-point Likert scale with the following response levels: 1 = completely disagree/stimme gar nicht zu/完全不同意, 2 = disagree/stimme nicht zu/不同意, 3 = rather disagree/stimme eher nicht zu/有一点不同意, 4 = rather agree/stimme eher zu/有点同意, 5 = agree/stimme zu/同意, 6 = completely agree/stimme voll und ganz zu/完全同意. Scoring of the IUES scales: *Positive reinforcement* is based on the mean score of the following items: 1, 3, 5, 7; *Avoidance expectancies* is based on the mean score of the following items: 2, 4, 6, 8.

**Table A3:** Internet Literacy Questionnaire (ILQ) in English, German, and Chinese language.

	English	German	Chinese
1.	I can quickly familiarize myself with new sites on the Internet or applications I have not used before.	Ich kann mich schnell mit neuen Internetseiten oder -anwendungen vertraut machen, die ich zuvor noch nicht benutzt habe.	我能够很快熟悉那些我从未使用过的网站和应用。
2.	It is easy for me to limit my Internet use when I notice that it has a negative effect on my private life.	Es fällt mir leicht, meinen Internetkonsum einzuschränken, wenn ich merke, dass er sich negativ auf mein Privatleben auswirkt.	当我察觉网络使用已经对我的个人生活产生负面影响时，我可以很容易地控制自己使用网络。
3.	It is easier to maintain social contacts on the Internet than offline.	Es ist einfacher im Internet soziale Kontakte zu pflegen als offline.	在网络中保持社交接触比线下更容易。
4.	I make sure to use the Internet to an extent that is appropriate for me.	Ich achte darauf, das Internet in einem für mich angemessenen Umfang zu nutzen.	我确保在一个对我适度的范围内使用网络。
5.	I can easily assess the credibility of information on websites even when I am not familiar with the topic.	Ich kann Informationen auf Internetseiten mühelos hinsichtlich ihrer Glaubwürdigkeit einschätzen, auch wenn mir das Thema nicht vertraut ist.	即便在不熟悉的领域里，我也可以比较容易地确定网站上的信息的可信程度。
6.	I inform myself regularly about developments regarding the Internet.	Ich informiere mich regelmäßig über Entwicklungen im Bereich des Internets.	我经常关注互联网发展方面的信息。
7.	When I am online, I make sure that my Internet use does not negatively affect my private life.	Wenn ich online bin, achte ich darauf, dass sich meine Internetnutzung nicht negativ auf mein Privatleben auswirkt.	当我在线的时候，我会注意我的网络使用情况不会对我的个人生活造成负面的影响。
8.	I notice very quickly when my counterpart on the Internet is pretending.	Ich merke sehr schnell, wenn sich mein Gegenüber im Internet verstellt.	当对方在网络上假意的时候，我很快可以注意到。
9.	Friends and acquaintances ask me for advice when they have problems with Internet applications.	Freunde und Bekannte fragen mich nach Rat, wenn sie Probleme mit Internetanwendungen haben.	当朋友们或熟人们遇到网络运用方面的问题时会请教我。
10.	Causally exchanging views with other people on the Internet is easier than offline.	Im Internet kann man sich zwangloser mit anderen Personen austauschen als offline.	在线上和他人随意地交换观点要比在线下更加容易。
11.	It is easier for me to make casual contact with another person online than offline.	Es fällt mir online leichter, unverbindlichen Kontakt mit einer anderen Person zu knüpfen als offline.	对我来说，与别人建立一般的联系在线上要比在线下更容易。
12.	I can easily assess what others on the Internet want to achieve with their behavior.	Ich kann gut einschätzen, was Andere im Internet mit ihrem Verhalten bezwecken wollen.	在网络中，我能比较容易地从他人的行为中推断出他们想要达到的目的。
13.	I can tell the difference between credible and untrustworthy content on the Internet.	Ich kann zwischen glaubwürdigen und unglaubwürdigen Inhalten im Internet unterscheiden.	我能在网络中分辨清楚可信和不可信的内容。
14.	It is easier for me to be creative online than offline.	Es fällt mir online leichter kreativ zu sein als offline.	对我来说，在线上比线下更容易有创造力。
15.	I go offline when I feel I have done or found everything on the Internet that is relevant at the time.	Ich gehe offline, wenn ich das Gefühl habe, alles gerade Relevante im Internet erledigt oder gefunden zu haben.	当我认为已经找到相关的信息或处理完相关的事情后，我就会下线。
16.	When I am on the Internet, I make sure not to be online longer than planned.	Wenn ich im Internet bin, achte ich darauf, dass ich nicht länger als beabsichtigt online bin.	当我使用网络时，我会确保不要超过计划的时间。
17.	I can deal with errors in computer software and apps and can fix them myself.	Ich kann mit auftretenden Fehlern in Computerprogrammen/Apps umgehen und sie eigenständig beheben.	我会自己处理和修理电脑软件和应用软件里的错误。
18.	I can more easily express myself on a topic on the Internet than offline.	Im Internet kann ich mich leichter zu einem Thema äußern als offline.	与线下相比，在网络上我更容易针对某一个话题发表自己的观点。

Items must be answered on a Likert scale of six response levels from 0 (=strongly disagree/stimme überhaupt nicht zu/非常不同意) to 5 (=strongly agree/stimme vollkommen zu/非常同意). Scoring of the ILQ scales: *Technical expertise* is based on the mean score of the following items: 1, 6, 9, 17; *Production & interaction* is based on the mean score of the following items: 3, 10, 11, 14, 18; *Reflection & critical analysis* is based on the mean score of the following items: 5, 8, 12, 13; *Self-regulation* is based on the mean score of the following items: 2, 4, 7, 15, 16.
