# Supplementary material for: Investigating the Effect of Personality, Internet Literacy, and Use Expectancies in Internet-Use Disorder: A Comparative Study between China and Germany
Source: Int J Environ Res Public Health. 2018 Mar 23;15(4):579. doi: 10.3390/ijerph15040579 (PMC5923621; doi:10.3390/ijerph15040579)
Supplement: Supplementary file 1 [file ijerph-15-00579-s001.pdf]

Investigating the Effect of Personality, Internet Literacy, and Use Expectancies in Internet-Use Disorder: A Comparative Study between China and Germany

Benjamin Stodt [1], Matthias Brand [1,2], Cornelia Sindermann [3], Elisa Wegmann [1], Mei Li [4], Min Zhou [5], Peng Sha [6] and Christian Montag [3,7,*]

1 General Psychology: Cognition and Center for Behavioral Addiction Research (CeBAR), University of Duisburg-Essen, 47057 Duisburg, Germany; benjamin.stodt@uni-due.de (B.S.); matthias.brand@uni-due.de (M.B.); elisa.wegmann@uni-due.de (E.W.)
2 Erwin L. Hahn Institute for Magnetic Resonance Imaging, 45141 Essen, Germany
3 Department of Molecular Psychology, Institute of Psychology and Education, Ulm University, 89081 Ulm, Germany; cornelia.sindermann@uni-ulm.de
4 Student Counselling Centre, Beijing University of Civil Engineering and Architecture, Beijing 100037, China; amorelm415@gmail.com
5 Institute of Medical Statistics, Informatics and Epidemiology, University of Cologne, 50923 Cologne, Germany; min.zhou@hotmail.de
6 School of Journalism and Communication, Southwest University, Chongqing 400716, China; a2352893@gmail.com
7 Key Laboratory for NeuroInformation/Center for Information in Medicine, School of Life Science and Technology, University of Electronic Science and Technology of China, Chengdu 611731, China
* Correspondence: christian.montag@uni-ulm.de; Tel.: +49-731-502-6550

Table S1. Gender differences in the s-IAT, IUES, ILQ, and Big Five personality traits in Germany and China (ANOVA).

	Germany				China							
	Male		Female		Male		Female					
**Domain/variable**	*M*	*SD*	*M*	*SD*	*M*	*SD*	*M*	*SD*	*F*	*df*	*p*	η^2
**Internet-use disorder (s-IAT)**												
Total score	24.81	7.48	23.35	7.38	31.53	8.89	31.08	10.30	0.71	1, 817	0.400	0.001
Loss of control/time management	14.41	4.39	13.80	4.76	16.97	4.69	17.31	5.38	1.91	1, 817	0.167	0.002
Craving/social problems	10.41	4.00	9.54	3.50	14.56	4.81	13.77	5.51	0.02	1, 817	0.900	<0.001
**Internet-use expectancies (IUES)**												
Positive reinforcement	3.96	1.12	3.35	1.22	4.37	0.88	4.21	0.96	8.98	1, 817	0.003	0.011
Avoidance expectancies	2.54	1.13	2.38	1.16	3.40	1.12	3.41	1.16	1.10	1, 817	0.295	0.001
**Internet literacy (ILQ)**												
Technical expertise	3.41	1.12	2.34	1.10	3.23	1.05	2.70	1.09	12.48	1, 817	<0.001	0.015
Production and interaction	2.24	1.09	1.96	1.13	3.01	1.04	2.79	1.15	0.22	1, 817	0.683	<0.001
Reflection and critical analysis	3.28	0.82	3.06	0.94	3.11	0.99	2.89	1.08	<0.01	1, 817	0.995	<0.001
Self-regulation	2.90	1.05	3.27	0.92	3.06	0.96	2.74	1.02	23.68	1, 817	<0.001	0.028

Table S1. *Cont.*

**Big Five personality**[1]												
Neuroticism	2.38	0.88	3.01	0.92	2.86	0.58	2.80	0.59	40.74	1, 817	<.001	.047
Extraversion	3.37	0.94	3.73	0.96	3.20	0.47	3.19	0.46	12.56	1, 817	<.001	.015
Openness	3.43	1.04	3.49	1.12	3.24	0.42	3.33	0.44	0.02	1, 817	.893	<.001
Conscientiousness	3.20	0.86	3.41	0.93	3.34	0.44	3.34	0.48	4.94	1, 817	.027	.006
Agreeableness	2.99	0.80	3.08	0.83	3.35	0.48	3.44	0.43	<0.01	1, 817	.947	<.001

[1]German sample: measured with BFI-10; Chinese sample: measured with NEO-FFI

Table S2. Correlations between age and the observed Internet and personality (Pearson correlations) including Fisher's *z* comparison between Germany and China.

	Age			
**Domain/variable**	**Total**	**Germany**	**China**	**Fisher's *z***
**Internet-use disorder (s-IAT)**				
Total score	.033	-.072	.142**	-3.07**
Loss of control/time management	.028	-.010	.074	-1.20
Craving/social problems	.033	-.129**	.191**	-4.61**
**Internet-use expectancies (IUES)**				
Positive reinforcement	.066	.096*	.020	1.09
Avoidance expectancies	.082*	.028	.164**	-1.96*
**Internet literacy (ILQ)**				
Technical expertise	.125**	.101*	.158**	-0.83
Production and interaction	.017	-.056	.110*	-2.38**
Reflection and critical analysis	.017	-.031	.070	-1.44
Self-regulation	.024	-.002	.059	-0.87
**Big Five personality**[1]				
Neuroticism	-.018	-.029	.003	-0.46
Extraversion	-.021	.013	-.109*	1.75*
Openness	-.144**	-.152**	-.149**	-0.04
Conscientiousness	.079*	.081	.080	0.01
Agreeableness	-.035	-.026	-.063	0.53

[1]German sample: measured with BFI-10; Chinese sample: measured with NEO-FFI
* $p \leq .05$, ** $p \leq .01$
